# Hydrogel–Flexible Electronics Integrated Platforms for Diabetic Wound Management

**DOI:** 10.3390/ma19030509

**Published:** 2026-01-27

**Authors:** Zhenjun Liu, Huanping Zhang, Yuqing Li, Shengxi Xu, Ning Fu, Fang Wang, Wansong Chen

**Affiliations:** 1 Henan Province Engineering Research Center of Key Materials and Technologies for Low-Altitude Aircraft Battery Systems, College of Chemistry and Environmental Engineering, Anyang Institute of Technology, Anyang 455000, China; liuzhenjun@ayit.edu.cn (Z.L.); 20160463@ayit.edu.cn (H.Z.); 2 Hunan Provincial Key Laboratory of Micro & Nano Materials Interface Science, College of Chemistry and Chemical Engineering, Central South University, Changsha 410083, China; osh1105913482@163.com; 3 School of Biomedical Engineering, The University of New South Wales, Sydney, NSW 2000, Australia; z5656600@ad.unsw.edu.au

**Keywords:** diabetic wounds, hydrogels, flexible electronics

## Abstract

Diabetic wounds are a major clinical challenge, driven by hyperglycemia, oxidative stress, persistent inflammation, and bacterial infection. Conventional dressings offer limited benefit, creating demand for advanced therapeutic strategies. This review analyzes hydrogel-based wound dressings and flexible electronic devices. Hydrogels are categorized by angiogenesis promotion, antioxidant activity, anti-inflammatory regulation, antibacterial action, and electrical conductivity. Flexible electronics are examined for adaptability, sensitivity, and real-time monitoring potential. Hydrogels maintain moist environments, support tissue regeneration, and deliver multifunctional bioactivity. Growth factor-loaded and electroactive hydrogels promote angiogenesis. Reactive oxygen species (ROS)-responsive systems restore redox balance. Anti-inflammatory and antibacterial hydrogels regulate macrophages and reduce infection risk. Conductive hydrogels accelerate healing through electrical stimulation. Flexible electronics provide continuous monitoring, intelligent feedback, and remote management, enhancing treatment precision. Their integration with hydrogels represents a paradigm shift from passive dressings to active diagnostic and therapeutic systems. Challenges remain in material design, interfacial stability, and long-term biocompatibility. These issues guide future innovation and clinical translation, offering a foundation for smart diabetic wound management.

## 1. Introduction

**Figure 1:**
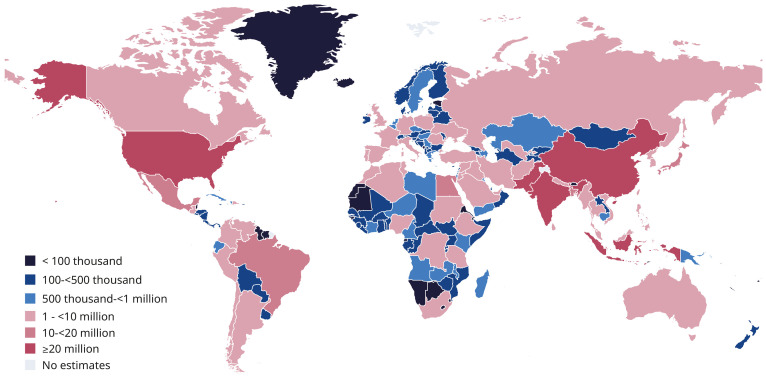
Global distribution of adults (20–79 years) with diabetes, 2024. Adapted with permission [7]. Copyright 2025, International Diabetes Federation.

Diabetes is defined as a chronic disease characterized by hyperglycemia. It represents an increasingly severe public health challenge with significant impacts on human health [1,2,3,4,5]. By the year 2045, it is estimated that the global population of individuals diagnosed with diabetes will reach 783 million. It is estimated that up to 25% of individuals will develop diabetic foot disease [6]. China currently has a substantial diabetic population, with tens of millions of patients diagnosed with diabetic foot disease (Figure 1) [7].

The term ‘diabetic foot’ encompasses pathologies including foot deformities, infections, and ulcerations, resulting from chronic diabetic neuropathy and vascular abnormalities in the lower limbs. Due to peripheral neuropathy with reduced pain sensation, minor injuries such as calluses and blisters often progress to ulcers without timely detection [8,9,10]. Persistent hyperglycemia severely disrupts wound healing. Compared to normal healing, diabetic wounds exhibit reduced endothelial cell proliferation and migration, impaired angiogenesis, and heightened inflammatory responses [11,12,13]. This healing impairment increases susceptibility to chronic ulcers, which may ultimately necessitate amputation ranging from toes to the lower leg. Traditional wound management relies on passive materials such as gauze and bandages, which primarily provide physical coverage and exudate absorption. These dressings lack dynamic responsiveness to the wound microenvironment and cannot intervene in the pathological processes that impede healing [14,15,16]. Consequently, there exists a demand for wound dressings that have the capability to absorb exudate while maintaining moisture levels within the wound, in addition to exhibiting multifunctional characteristics. These include antibacterial properties, which can contribute to the healing process for wounds afflicted with diabetes.

Recent years have seen a paradigm shift in chronic wound management, driven by integrating functionalized hydrogels with flexible electronics. Hydrogels, defined by high water content, three-dimensional networks, biocompatibility, and tunable mechanical properties, have emerged as optimal wound dressing materials [17,18]. Through molecular modifications and responsive design, they can deliver multiple therapeutic functions including antibacterial [19], antioxidant [20,21], and pro-angiogenic effects [22,23]. Concurrently, flexible electronic devices, distinguished by mechanical flexibility and high sensitivity, enable real-time wound monitoring, intelligent feedback, and remote interaction. The synergistic integration of these technologies enhances treatment precision and timeliness, driving a transition from passive dressings to active diagnostic and therapeutic systems.

Nevertheless, the utilization of integrated hydrogel–flexible electronics systems continues to encounter substantial challenges with respect to their material composition, interfacial stability, and systemic integration. The following key research topics have been identified: the synergistic design of multifunctional materials, stable interfacial signal transmission, and long-term biocompatibility. This work provides a systematic review of the integration of functionalized hydrogels and flexible electronics from a materials science perspective, exploring their potential applications and the engineering constraints that exist in the management of diabetic wounds. It also outlines prospective future directions to provide a theoretical foundation to support technological innovation and clinical translation in this field.

## 2. Challenges in Diabetic Wound Healing

### 2.1. Normal Wound Healing Process

**Figure 2:**
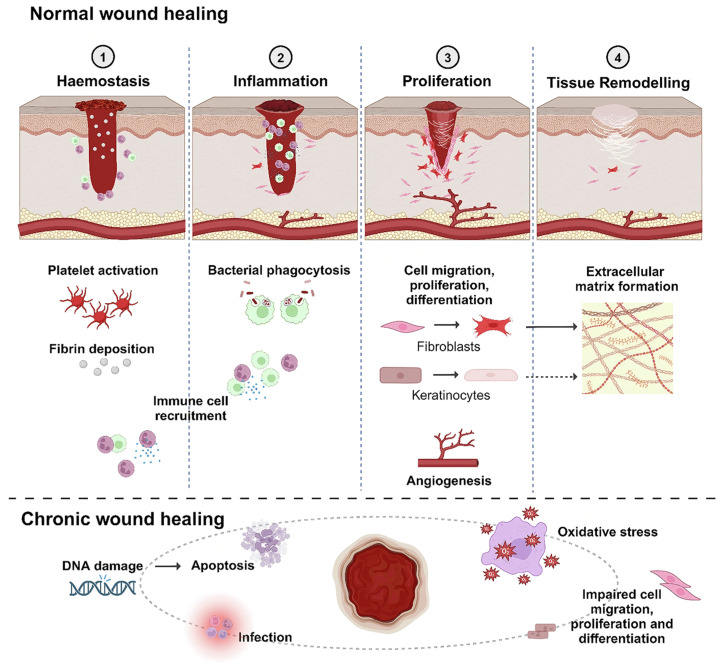
The normal wound healing process: ① bleeding and hemostasis, ② inflammation, ③ proliferation, and ④ remodeling [25].

Normal skin wound healing is a dynamic and highly coordinated biological process that primarily consists of four sequential yet overlapping stages: hemostasis, inflammation, proliferation, and tissue remodeling (as illustrated in Figure 2). These stages interact in a temporally regulated manner, making skin wound healing one of the most intricate regenerative processes in the human body [24,25].

Following integumentary injury, initial physiological responses involve vasoconstriction and platelet activation at the wound site to minimize hemorrhage and initiate fibrin clot formation. Inflammatory cells such as neutrophils and macrophages are subsequently recruited to the clot, serving functions including antimicrobial defense, debris clearance, and stimulation of cell proliferation and angiogenesis. Upon resolution of inflammation, endothelial cells near the wound proliferate and migrate, forming new blood vessels that deliver nutrients and oxygen essential for tissue regeneration. Fibroblasts then invade the clot to form granulation tissue while re-epithelialization proceeds; as epithelial cells proliferate and migrate across the wound bed, healing nears completion, typically restoring skin barrier function and tensile strength [26,27].

### 2.2. Wound Healing Process in Diabetes

Diabetic patients are particularly susceptible to chronic or non-healing wounds as a result of dysregulated blood glucose levels. Although the precise pathogenesis of diabetic wounds remains incompletely understood, current evidence suggests the involvement of multiple intrinsic and extrinsic factors. Existing research has identified several key contributors, including hyperglycemic toxicity [28], the accumulation of ROS induced by oxidative stress [29,30], sustained and excessive inflammatory cell infiltration [31], and recurrent bacterial infections [32]. It is evident that these elements contribute to an elongation of the four customary stages of normal wound healing, thereby impeding the healing process of diabetic wounds and leading to their progression into chronic wounds [12].

#### 2.2.1. Hyperglycemia

Hyperglycemia, a common pathological state in diabetes, impairs wound healing through multiple mechanisms. Elevated glucose in the wound microenvironment reduces macrophage infiltration, compromising the inflammatory phase essential for healing initiation [33,34]. Under hyperglycemic conditions, glucose undergoes non-enzymatic glycation with proteins, forming advanced glycation end products (AGEs). AGEs induce collagen glycation, creating cross-linked structures that reduce extracellular matrix hydrophilicity, thereby impairing angiogenesis and granulation tissue formation and ultimately prolonging healing [33,34].

#### 2.2.2. Accumulation of Reactive Oxygen Species

Normal wound healing involves redox signaling in which physiological ROS levels promote fibroblast activity, extracellular matrix synthesis, and angiogenesis via VEGF upregulation [35,36,37]. Maintaining optimal ROS concentrations is thus essential for healing and tissue homeostasis. However, diabetic wounds exhibit elevated ROS associated with hyperglycemia [38]. This accumulation stems from impaired glycolytic metabolism and AGE formation. AGEs induce glycation of mitochondrial respiratory chain proteins, resulting in excessive production of ROS (e.g., H_2_O_2_ and ·O_2_^−^). These elevated ROS species then activate NADPH oxidase, amplifying oxidative stress while simultaneously promoting glucose oxidation. This dual action accelerates further AGE formation, establishing a self-perpetuating deleterious cycle that impairs healing in diabetic wounds [39,40,41].

#### 2.2.3. Inflammatory Infiltration

**Figure 3:**
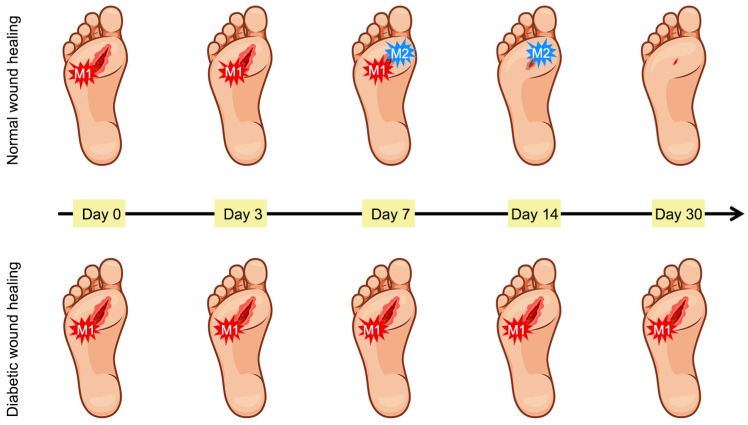
Macrophage phenotypes in normal and diabetic wounds.

Persistent inflammatory infiltration characterizes diabetic wounds, where dysregulated cytokine balance impairs healing [42]. Chronic tissue damage and repair failure drive excessive accumulation of immune cells, particularly neutrophils and macrophages, perpetuating a pro-inflammatory cytokine cascade [34]. A central mechanism underlying this persistent inflammatory state is the disrupted phenotypic transition of macrophages from the pro-inflammatory M1 state to the anti-inflammatory M2 phenotype (as shown in Figure 3) [43,44]. During the normal process of wound healing, M1 macrophages predominate in the initial phase (approximately 3 days), subsequently transitioning to M2 macrophages. These M2 macrophages release angiogenesis-related factors and anti-inflammatory cytokines, which promote vascular regeneration. In the context of diabetic wounds, however, macrophages characteristically sustain an M1 phenotype, thereby perpetuating a state of inflammatory infiltration [45]. Furthermore, diabetes promotes excessive neutrophil extracellular trap production. Although these traps contribute to pathogen clearance and debridement, their overproduction damages epithelial and endothelial cells, delaying healing [46,47].

#### 2.2.4. Bacterial Infection

Bacterial infection significantly impairs chronic diabetic wound healing [48]. While intact epidermis efficiently resists contamination, disrupted healing in diabetic wounds creates portals for colonization [49]. Diabetic wounds, characterized by persistently elevated glucose levels in the wound microenvironment, provide a particularly favorable niche for bacterial proliferation. Adherent bacteria often form structured biofilms that facilitate quorum-sensing communication and enhance survival through physical protection and antibiotic resistance. Biofilms also stimulate localized inflammatory responses that further promote biofilm development, establishing a self-reinforcing cycle of infection and inflammation that contributes to wound chronicity [50,51].

## 3. Hydrogel-Based Diabetic Wound Therapy

Hydrogels are regarded as optimal materials for the treatment of diabetic wounds. Hydrogels are hydrophilic three-dimensional polymer networks. They exhibit excellent swelling properties, which provide a long-term stable moist environment during wound repair, thereby facilitating granulation tissue growth. The mechanical properties of these materials are comparable to those of soft tissue extracellular matrices, and their excellent biocompatibility supports wound tissue regeneration and re-epithelialization. As a consequence, hydrogels are regarded as the most competitive dressing material available at the present time. In recent years, there has been a notable increase in research activity focusing on the design and preparation of advanced hydrogel wound dressings [52,53,54].

### 3.1. Angiogenesis-Promoting Hydrogel Wound Dressings

In diabetic patients, hyperglycemia-induced endothelial dysfunction has been shown to impair angiogenesis and compromise vascular integrity, ultimately resulting in delayed or aberrant wound healing. Accordingly, the effective stimulation of angiogenesis holds substantial therapeutic value in the management of diabetic wounds [55,56]. Based on their angiogenic mechanisms and structural design strategies, current angiogenic hydrogels can be broadly categorized into two types: those loaded with growth factors and those incorporating pro-angiogenic materials.

Exogenous growth factor delivery has been extensively demonstrated to promote angiogenesis and accelerate wound repair. Among these, VEGF plays a pivotal role in revascularization, a therapeutic approach aimed at enhancing oxygen supply within wound sites. This insight has spurred the investigation of VEGF-based therapies for diabetic wound treatment [57]. However, direct protein administration faces several limitations, including poor bioavailability of macromolecular proteins, rapid degradation, and high production costs, which collectively hinder clinical efficacy [58]. To overcome VEGF instability and deficiency under the oxidative and inflammatory conditions characteristic of diabetic wounds, hydrogels have been employed as sustained-release platforms. These hydrogel matrices act as protective reservoirs, enabling controlled and prolonged release of bioactive proteins, thereby maintaining therapeutic VEGF levels over time. For instance, Zhao et al. developed a supramolecular hydrogel patch with robust mechanical properties by combining a temperature-responsive poly(N-isopropylacrylamide) filler with a mixed supramolecular scaffold composed of N-acryloylglycine and 1-vinyl-1,2,4-triazole [59]. Under elevated temperatures associated with inflammatory responses, this hydrogel system exhibited temperature-triggered release of active agents. Such responsiveness was shown to facilitate re-epithelialization and enhance angiogenesis within the granulation tissue of diabetic wounds.

Emerging evidence suggests that, beyond their role in promoting cellular proliferation, certain biomaterials possess intrinsic capabilities to regulate the stability, bioactivity, and spatial distribution of cytokines and growth factors. The incorporation of such biomaterials into therapeutic platforms not only alleviates concerns regarding drug degradation and bioavailability but also enables direct modulation of local tissue repair and regeneration processes. For example, Li et al. developed a multifunctional hydrogel based on L-arginine-conjugated chitosan, harnessing the pro-angiogenic properties of L-arginine alongside the tissue-repairing functions of chitosan [34]. Their study demonstrated that this hydrogel effectively promoted re-epithelialization, collagen deposition, and angiogenesis in diabetic wound models. In addition to the intrinsic bioactivity of the constituent materials, specific physicochemical properties of hydrogels such as electrical conductivity and piezoelectricity can be strategically utilized to further promote angiogenesis [60,61]. Reports have shown that electric fields can stimulate endothelial cell proliferation and upregulate the expression of VEGF, thereby activating downstream signaling cascades essential for neovascularization during wound healing [62].

Building upon this principle, Wang et al. synthesized a self-powered electroactive composite hydrogel based on polyvinyl alcohol (PVA) [61]. Dipole interactions between PVA and polyvinylidene fluoride (PVDF) chains facilitated the formation of the electroactive β phase of PVDF. The piezoelectric properties of PVDF enabled the conversion of mechanical energy generated by rat movement into electrical energy, which in turn amplified the endogenous electric field within diabetic wound sites. This bioelectrical stimulation was shown to significantly enhance cellular proliferation, migration, and angiogenesis.

### 3.2. Antioxidant Hydrogel Wound Dressings

**Figure 4:**
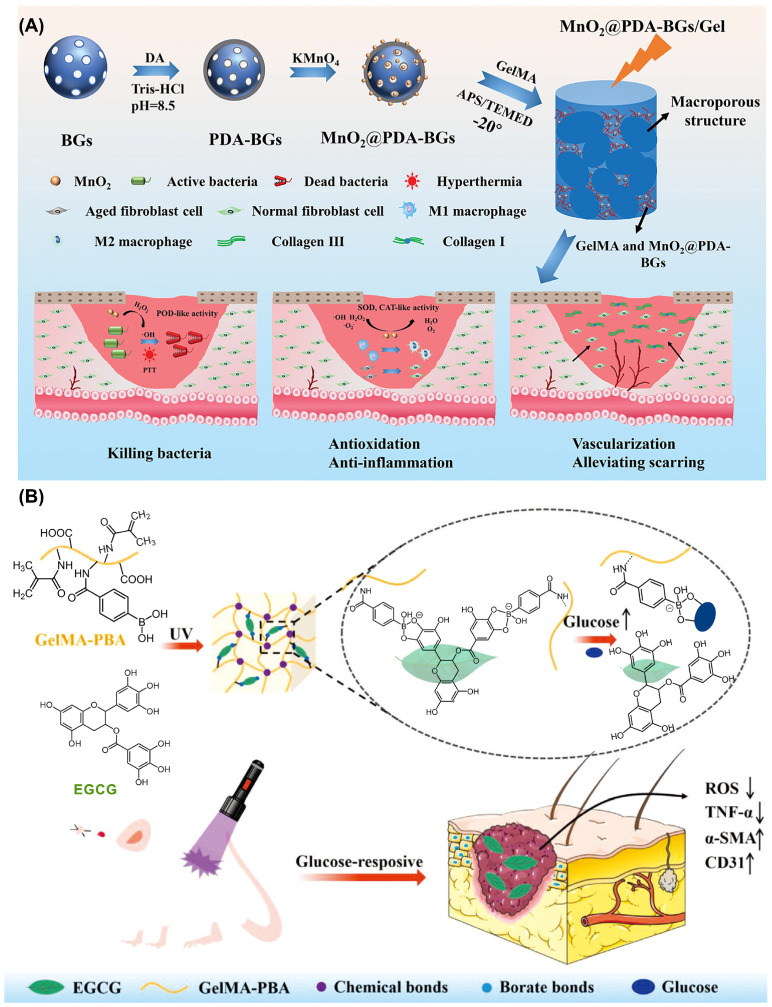
(**A**) Schematic illustration of MnO_2_@PDA-BG hydrogel synthesis and diabetic wound treatment. Adapted with permission [66]. Copyright 2023, Wiley-VCH. (**B**) Schematic of the GelMA-CPBA/EGCG hydrogel synthesis and its application in diabetic wound treatment via ROS scavenging for anti-inflammation. Adapted with permission [67]. Copyright 2023, Wiley-VCH.

Diabetic wounds often show an imbalance between oxidative stress and antioxidant defense. This condition results in chronically elevated ROS, disrupted cellular metabolism, and accelerated senescence and apoptosis. Given the dynamic fluctuations in ROS concentrations at the wound site, modulating hydrogel degradation in response to ROS levels has emerged as a promising strategy for achieving intelligent and responsive therapeutic interventions in diabetic wound care [63]. The following section outlines current methodologies for the construction of antioxidant hydrogels. A critical step in this process involves grafting antioxidant moieties onto the hydrogel matrix. Additionally, antioxidant components may be incorporated into the hydrogel polymer chains through polymerization or grafting reactions [64,65]. For instance, Cao et al. introduced polydopamine (PDA) coated manganese dioxide (MnO_2_), known for its antioxidant activity, into a hydrogel system to synthesize a bioactive glass (BG)-based nanocomposite hydrogel (MnO_2_@PDA-BGs), as illustrated in Figure 4A [66]. The superoxide dismutase-like and catalase-like enzymatic properties of MnO_2_ facilitate the conversion of hydrogen peroxide (H_2_O_2_) and superoxide anions (·O_2_^−^) into water and oxygen, thereby mitigating oxidative stress and alleviating hypoxic conditions at the wound site. In a complementary approach, Sun et al. developed an antioxidant hydrogel using a different design strategy, as shown in Figure 4B [67]. In this system, methacryloyl gelatin (GelMA) was graft-modified with 4-carboxyphenylboronic acid (CPBA) and subsequently photopolymerized with (-)-epigallocatechin gallate (EGCG) to form the GelMA-CPBA/EGCG hydrogel. The phenylboronic acid (PBA) groups of GelMA-CPBA interact with the ortho-dihydroxyl groups of EGCG to form glucose-responsive borate ester bonds. Under hyperglycemic conditions typical of diabetic wounds, these bonds undergo cleavage, triggering the release of EGCG. The released EGCG acts as a natural antioxidant, effectively scavenging excess ROS and contributing to the restoration of redox balance.

### 3.3. Anti-Inflammatory Hydrogel Wound Dressings

**Figure 5:**
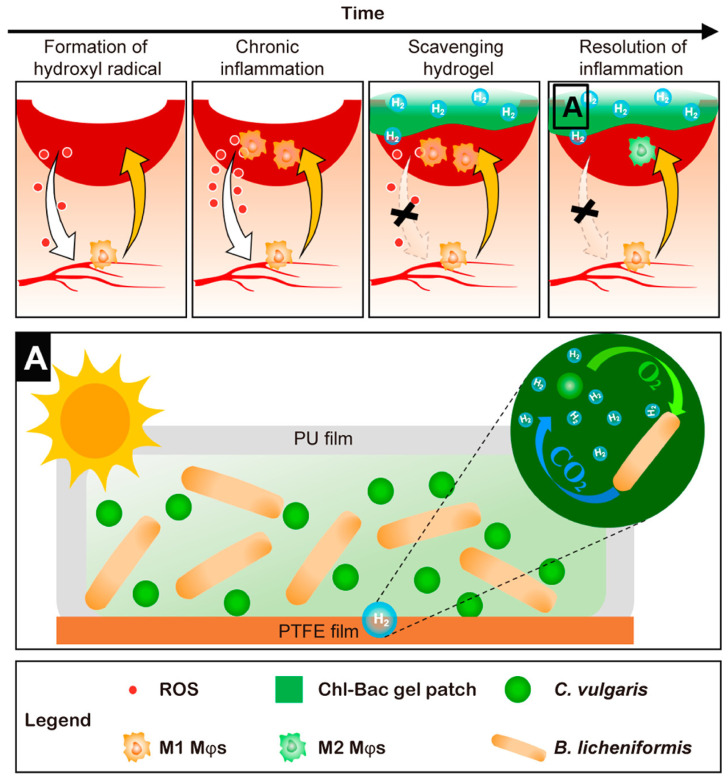
Schematic mechanism of hydrogen in diabetic wound therapy. Adapted with permission [72]. Copyright 2022, American Chemical Society.

Macrophages play a pivotal bridging role in both the inflammatory and proliferative phases of wound healing. Accordingly, most anti-inflammatory hydrogel wound dressings are designed to regulate macrophage polarization, promoting the transition from the pro-inflammatory M1 phenotype to the anti-inflammatory M2 phenotype [68,69]. For instance, Jiang et al. developed a *B. striata* polysaccharide methacrylate (BSPMA)-GelMA hydrogel that effectively modulates macrophage phenotypes. This hydrogel was shown to suppress the expression of the pro-inflammatory cytokine tumor necrosis factor-alpha while upregulating the anti-inflammatory cytokine interleukin-10 in diabetic wound models, indicating its considerable therapeutic potential [70]. Fu et al. further advanced this concept by designing a three-dimensional porous polyurethane and hyaluronic acid hybrid hydrogel (PUHA-RGD), which was inspired by the structural features of the extracellular matrix. This hydrogel downregulates genes associated with cytokine and cytokine receptor interactions, thereby inducing reprogramming of the immune microenvironment. Both in vitro and in vivo experiments confirmed its ability to regulate macrophage polarization, suppress inflammation, and promote wound healing in diabetic models [71]. To overcome the limitations associated with conventional hydrogen therapy, Wu and colleagues engineered a hydrogel patch incorporating live algae and bacteria (Figure 5). Under hypoxic conditions, chlorella algae embedded within the patch utilize solar energy to synthesize biohydrogen. The generated hydrogen acts as an anti-inflammatory agent, reprogramming M1 macrophages at diabetic wound sites into M2 macrophages, thereby restoring a wound microenvironment conducive to healing [72].

### 3.4. Antibacterial Hydrogel Wound Dressings

Due to compromised immune function, diabetic patients exhibit a reduced capacity to resist bacterial infections and therefore require external antimicrobial interventions [73]. The classification of antimicrobial hydrogels is primarily based on their construction strategies and underlying antimicrobial mechanisms. These hydrogels can be broadly categorized into three types: (1) hydrogels loaded with antibiotics, (2) hydrogels incorporating inorganic nanoparticles, and (3) hydrogels possessing intrinsic antimicrobial properties [74,75,76].

Currently, antibiotics remain the most widely used antimicrobial agents in clinical practice, and antibiotic-loaded hydrogel wound dressings have attracted considerable research attention. For example, Malkoch et al. developed a dual-antibiotic delivery hydrogel dressing with tunable mechanical properties. This system incorporates two active pharmaceutical ingredients: hydrophilic neomycin sodium and hydrophobic ciprofloxacin. The hybrid design enables rapid release of neomycin sodium while sustaining the release of ciprofloxacin, thereby enhancing overall antimicrobial efficacy [77]. Despite their effectiveness, prolonged antibiotic use may lead to the development of bacterial resistance, which poses a significant challenge in the management of diabetic wounds [78,79]. To address this issue, increasing attention has been directed toward inorganic nanomaterial-based antimicrobial strategies. Incorporating inorganic nanoparticles into hydrogel matrices has been shown to improve antimicrobial performance and maintain long-term antibacterial activity, thereby mitigating the risk of resistance development. Among these, silver nanoparticles are the most extensively studied and applied [80]. For instance, Xiao et al. developed an injectable collagen–silver nanoparticle hydrogel, leveraging the broad-spectrum antibacterial activity of silver nanoparticles against multidrug-resistant bacteria. This hydrogel demonstrated potent antibacterial efficacy and exhibited promising potential for promoting wound regeneration [81]. In addition to externally loaded agents, the antimicrobial properties of hydrogels can also arise from the intrinsic characteristics of their precursor materials. Cationic polysaccharides such as chitosan, commonly used as hydrogel precursors, possess inherent antibacterial activity. This is primarily achieved through electrostatic interactions with negatively charged bacterial membranes or biomolecules, leading to membrane disruption and interference with bacterial metabolism [82,83].

### 3.5. Conductive Hydrogel Wound Dressings

**Figure 6:**
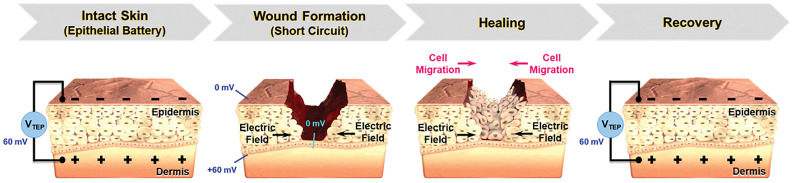
Trans-epithelial potential and electric field at the wound site before and after the healing process. Adapted with permission [88]. Copyright 2020, Wiley-VCH.

As early as 1969, a series of reports indicated that electrical stimulation exerted a beneficial effect on wound healing processes [84]. Subsequent clinical studies have demonstrated that electrical stimulation can induce re-epithelialization in both skin and corneal wounds by promoting the migration and proliferation of fibroblasts, keratinocytes, and epithelial cells, while concurrently reducing edema formation. These findings underscore the therapeutic potential of electrical stimulation in the fields of skin tissue engineering and wound management [85,86]. Directed cell migration is a critical component of effective wound healing. Electrotaxis, defined as the directional movement of cells in response to electric fields, is a complex and cell type-specific phenomenon. At wound sites, both endogenous and externally applied electric fields can guide cellular migration and thereby facilitate tissue repair (Figure 6) [87,88].

In recent years, conductive hydrogels with high electrical conductivity and excellent biocompatibility have been developed to address the limitations of conventional metallic electrodes, which often fail to achieve conformal contact with irregular wound surfaces due to mechanical mismatch. These hydrogels enable efficient delivery of electrical stimulation directly to the wound bed. For example, Li et al. designed an ion-conductive hydrogel that, under exogenous electric field stimulation, was shown to promote macrophage polarization, stimulate angiogenesis, and enhance collagen deposition in diabetic wound models [89]. Beyond serving as passive carriers for external stimulation, conductive hydrogels can also act as active conduits for endogenous electrical signals. Their intrinsic conductivity facilitates intercellular communication and supports the directional migration of epithelial cells, thereby promoting re-epithelialization and accelerating wound closure [90].

## 4. Flexible Electronic Devices

### 4.1. Overview of Flexible Electronic Devices

**Figure 7:**
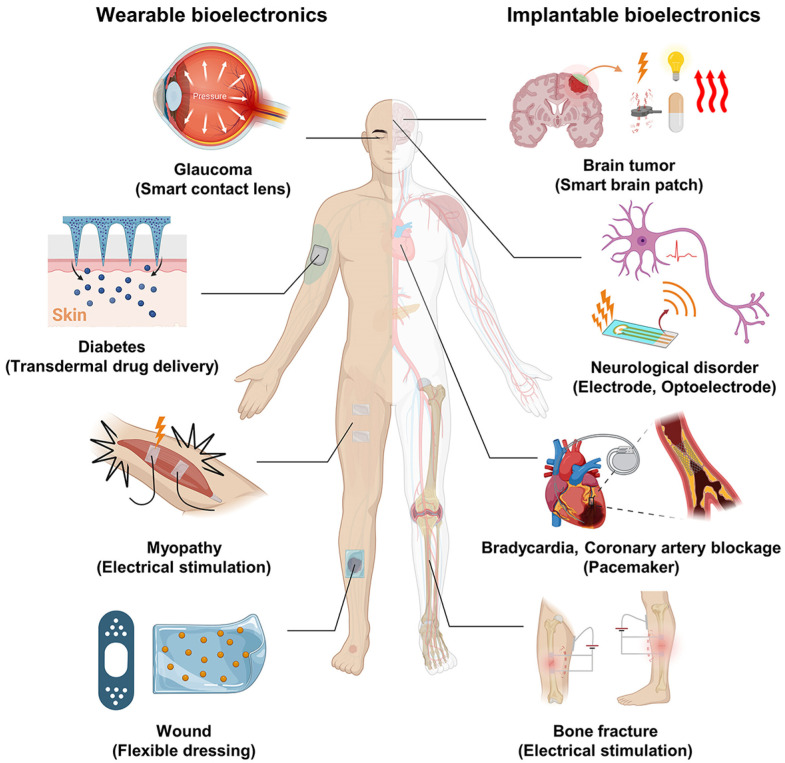
Applications of flexible electronics in medical fields. Adapted with permission [93]. Copyright 2023, American Chemical Society.

The integration of electronic devices into human life has been significantly accelerated by continuous advancements in high-tech innovation. The development of emerging electronic technologies, such as electronic skin, nanogenerators, brain–computer interfaces, and implantable medical devices, remains an active area of research and application. Compared with traditional rigid electronics based on silicon, which are increasingly constrained by the limitations of Moore’s Law, flexible electronics offer a range of advantageous properties. These include reduced thickness, lightweight structure, low mechanical modulus, and the ability to stretch. Such features allow flexible electronics to conform seamlessly to various surfaces, rendering them effectively imperceptible in mechanical terms. These devices can be directly attached to the skin, integrated into garments, or implanted within the body, corresponding to epidermal electronics, wearable electronics, and implantable electronics, respectively. Owing to their mechanical adaptability, wearability, and compatibility with diverse application scenarios, flexible electronics have found widespread use in health monitoring systems, artificial electronic skin, and energy harvesting or storage platforms (Figure 7) [91,92,93].

In the medical field, flexible electronics provide an optimal platform for patient self-management and personalized healthcare, as illustrated in Figure 7. This technology enables non-invasive, continuous monitoring of vital physiological signals in real time and in situ, thereby enhancing patient comfort and compliance. The data obtained through these systems are directly relevant to clinical decision-making, particularly in the context of preventive care and early disease diagnosis [94]. Moreover, flexible electronics have demonstrated significant advantages in the long-term tracking and management of chronic health conditions, including diabetes, cardiovascular diseases, and metabolic disorders. This capability is especially important for elderly individuals and patients suffering from persistent illnesses [95]. In the context of diabetic wound care, flexible electronics can be broadly categorized into two functional types based on their primary roles: devices designed for the detection of diabetic wounds and devices engineered for therapeutic intervention.

### 4.2. Flexible Electronic Devices for Diabetic Wound Monitoring

Real-time monitoring and assessment of wound status are essential for the effective diagnosis and management of diabetic wounds. However, current clinical practices for wound evaluation predominantly rely on the intermittent removal of dressings to perform visual inspections or wound culture tests [96]. This limitation underscores the urgent need for the development of advanced medical devices tailored for wound management. The adoption of intelligent monitoring systems by clinicians enables continuous evaluation of wound healing dynamics, thereby facilitating the timely adjustment and optimization of therapeutic strategies. In the domain of flexible electronics, researchers have dedicated considerable effort to the development of diagnostic flexible electronic devices capable of recording alterations in various wound biomarkers, including temperature [97], pH [98], glucose [99] and lactate, etc. [100]. For example, Amay J. Bandodkar and colleagues designed a miniature, wireless, battery-free wound monitoring device capable of measuring lactate concentrations at the wound site in real time [100]. Lactate plays a critical role in initiating the wound healing cascade, with physiological levels typically ranging from 5 to 15 millimoles per liter (mM). Deviations from this range have been shown to impair healing: elevated lactate levels can induce severe hypoxia and delay tissue repair, whereas insufficient lactate levels may hinder the initiation of the healing process. By continuously monitoring lactate concentrations in diabetic mouse wound models and analyzing the resulting data, the researchers achieved an 83% accuracy rate in predicting wound closure outcomes.

**Figure 8:**
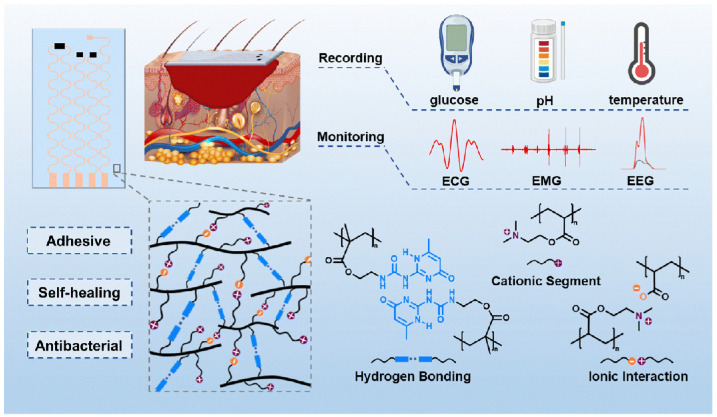
Schematic diagram of a monitored diabetic wound dressing. Adapted with permission [102]. Copyright 2024, Royal Society of Chemistry.

As research has progressed, flexible electronic devices capable of simultaneously monitoring multiple wound parameters have also been developed [101]. For instance, Huang et al. introduced a diagnostic wound dressing (Figure 8) designed for long-term diabetic wound management [102]. The hydrogel substrates demonstrated strong adhesion, self-healing capacity, and antibacterial activity, arising from abundant hydrogen bonding, ionic interactions, and cationic chain segments (Figure 8). This system integrates multiplexed sensors for the concurrent detection of key wound biomarkers, such as glucose, pH, and temperature, across their clinically relevant ranges, thereby enabling comprehensive documentation of the healing progress. In addition to biochemical monitoring, the smart dressing also supports real-time acquisition of electrophysiological signals, such as electrocardiograms, electromyograms, and electroencephalograms. These data streams provide clinicians with valuable insights into the patient’s overall health status and assist in clinical decision-making.

Recent advancements have pushed the boundaries of wound monitoring toward more integrated and intelligent systems. For instance, multimodal sensing platforms now combine various sensing mechanisms within a single device. A notable example is a battery-free, multifunctional microfluidic Janus wound dressing (MM-JWD) that integrates multiple colorimetric sensors for simultaneous monitoring of temperature, pH, and uric acid at the wound site [103]. This system employs smartphone-based image analysis for remote, real-time assessment of wound status, representing a significant step forward in point-of-care wound diagnostics. Another innovative approach involves microgel-based sensing dressings that utilize cholesterol liquid crystals, pH indicators, and glucose-sensitive enzymes encapsulated within hydrogel microspheres [104]. These “pixelated” sensors provide visual colorimetric feedback on wound temperature, pH, and glucose levels, which can be quantified through smartphone RGB analysis. This technology enables continuous monitoring without frequent dressing changes, offering a practical solution for long-term wound management.

For specialized monitoring applications, photoacoustic sensing systems have emerged as a promising technology. Researchers have developed a wearable PVA/sucrose hydrogel patch incorporating nitrazine yellow, a pH-responsive dye with excellent photoacoustic properties [105]. When combined with a portable, miniaturized photoacoustic analysis device, this system can accurately detect wound pH across a wide and clinically relevant range, unaffected by blood color interference—a significant advantage over traditional colorimetric methods. Additionally, large-scale, high-resolution temperature mapping has been achieved through flexible amorphous silicon temperature sensor arrays with high spatial resolution and sensitivity [106]. These arrays enable dynamic localization of inflammation regions across large wound areas, providing detailed thermal maps that correlate with healing progression and infection status.

### 4.3. Flexible Electronic Devices for Diabetic Wound Treatment

**Figure 9:**
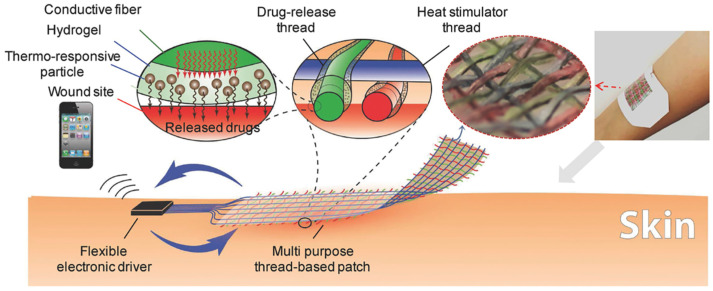
Schematic diagram of a drug delivery textile dressing based on a flexible threaded microheater. Adapted with permission [110]. Copyright 2017, Wiley-VCH.

Compared with wound monitoring alone, accelerating the healing of diabetic wounds through pharmaceutical intervention holds greater clinical significance. The incorporation of flexible electronic devices into wound monitoring systems offers a distinctive strategy for the active management of diabetic wounds. To achieve precise drug delivery and enable on-demand therapeutic responses, extensive research has focused on integrating flexible electronics into smart wound dressings [107]. Currently, thermoregulation represents the predominant approach in this domain, primarily utilizing thermoresponsive materials such as hydrogels as drug carriers. Temperature modulation via electronic components has been shown to enable controlled and timely release of therapeutic agents [108,109]. For example, Khademhosseini et al. developed a textile-based drug delivery system incorporating flexible thread microheaters (Figure 9). In this design, drug-loaded thermoresponsive hydrogels were coated onto specially engineered thermally conductive fibers, which were subsequently woven into fabric using textile manufacturing techniques. Upon electrical activation, the fabric facilitated targeted drug release tailored to the specific needs of diabetic wound environments [110]. Beyond temperature regulation, additional strategies such as electrical stimulation [111] and magnetoelectric stimulation [112] have also been explored.

**Figure 10:**
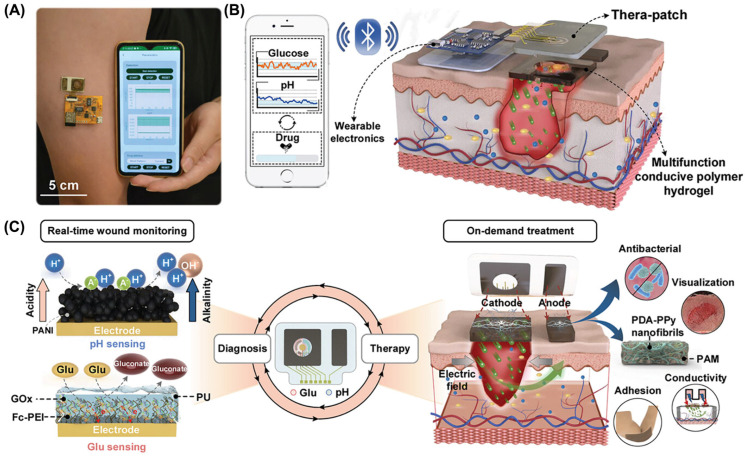
Schematic of a wearable device for wireless diagnostic and therapeutic integration for diabetic wound management. (**A**) Photograph of the integrated system. (**B**) Schematic showing the smartphone app, wearable device, and Thera-patch for monitoring and therapy. (**C**) Closed-loop strategy for real-time sensing and on-demand insulin delivery. Adapted with permission [114]. Copyright 2024, Wiley-VCH.

In recent years, growing attention has been directed toward the development of fully integrated closed-loop systems that combine wound biomonitoring with therapeutic delivery. These systems aim to achieve active, minimally invasive treatment of diabetic wounds. Therapeutic flexible electronics have emerged as a promising alternative for managing chronic diseases, offering real-time, continuous, high-quality, and responsive treatment in a controllable and user-friendly format [93,113]. As a representative example, Jiang et al. designed a fully integrated wearable flexible electronic device capable of wireless monitoring and active therapy (Figure 10). This system comprises three core components: a customized mobile application, wearable electronic modules, and a therapeutic patch. The patch is fabricated from a multifunctional conductive polymer hydrogel, prepared by embedding polydopamine–polypyrrole (PDA-PPy) nanofibrils into a polyacrylamide (PAM) network through an in situ process. Benefiting from its unique architecture and the polycationic backbone of PDA-PPy nanofibrils, the hydrogel exhibits high drug-loading efficiency, optical transparency, strong skin adhesion, electrical conductivity, and broad-spectrum antimicrobial activity. In addition, the working electrode for glucose sensing was functionalized with dendrimers incorporating the electron mediator ferrocene-cored poly(ethylenimine) (Fc-PEI) and glucose oxidase (GOx), thereby enhancing the sensitivity and selectivity of biomarker detection. By integrating smart hydrogel materials with wearable bioelectronic platforms, the system enables real-time monitoring of wound biomarkers such as pH and glucose. Furthermore, the device supports electrical stimulation and iontophoresis-based insulin delivery, thereby achieving comprehensive and on-demand therapeutic intervention [114].

The field has witnessed remarkable innovations in self-powered therapeutic systems that eliminate the need for external batteries. A prominent example is the flexible self-powered bandage based on PVA/Ecoflex/MXene/borate/glycerol hydrogel, which demonstrates excellent power output and maintains performance under extreme conditions [87]. This bandage serves dual functions as a motion sensor for human activity monitoring and as an electrical stimulation device for accelerating diabetic wound repair through enhanced cell proliferation, collagen deposition, and angiogenesis. Similarly, triboelectric nanogenerators (TENGs) have been integrated into wound dressings to convert biomechanical energy into therapeutic electrical pulses. Research on bilateral flexible TENG-based hydrogel skin patches has shown that chitosan/PVA/ZnO nanoparticle hydrogels combined with TENG-generated electrical stimulation significantly promote wound healing, with slower electrical pulses proving more effective than rapid ones [115].

Advanced closed-loop therapeutic platforms represent the cutting edge of integrated wound management. One innovative system combines a wearable pulse piezoelectric nanogenerator (PENG) with a phosphatase and tensin homolog (PTEN) inhibitor-loaded electroresponsive hydrogel [116]. This setup converts animal movement into an electric field that simultaneously stimulates wound tissue and triggers on-demand drug release when the field intensity exceeds a defined threshold. This approach enhances cellular electrotaxis while avoiding the negative effects of sustained PTEN inhibition. Another breakthrough is a wireless, programmable, refillable hydrogel bioelectronic device that integrates sensing, electrical stimulation, and controlled drug release (e.g., metformin) within a single platform [117]. This system demonstrated accelerated wound healing in diabetic rat models over 21 days, showcasing the potential of programmable electroceutical interventions.

For more targeted interventions, microneedle-based bioelectronic systems have shown exceptional promise. Inspired by beetle antennae, researchers developed a wearable, battery-free, wireless intelligent dendritic rivet-like multilayer hydrogel electroactive microneedle bioelectronic device (GP-eMN) [118]. This system enables precise drug delivery, TENG-driven electrical stimulation, and visual assessment of wound conditions through simultaneous detection of uric acid, pH, and glucose. The bioinspired structure ensures mechanical interlocking with the dermis for effective, sustained drug delivery and rapid interstitial fluid uptake.

### 4.4. Integrated Hydrogel–Electronics Platforms for Theranostics

The most significant advancements in smart wound management emerge from deep material and functional integration, where hydrogels and flexible electronics are not merely combined but are engineered as synergistic components of unified therapeutic systems. This convergence creates platforms with capabilities exceeding the sum of their parts, enabling unprecedented levels of intelligent wound management.

#### 4.4.1. Conductive and Responsive Hydrogel Architectures

At the foundation of integrated systems lies the development of hydrogels with intrinsic electronic functionality. Rather than attaching separate electrodes, researchers are engineering hydrogels that themselves serve as conductive elements. For instance, heparin-dopamine conjugated hydrogels incorporating reduced graphene oxide (Hep-PDA-rGO) achieve high conductivity while maintaining excellent biocompatibility [119]. These materials function simultaneously as epidermal strain sensors and active wound dressings, promoting angiogenesis through electrical conductivity while providing antibacterial and antioxidant effects. Similarly, Ag@polydopamine-functionalized boronate-linked chitosan hydrogels demonstrate multifunctional capabilities including strain sensing, rapid self-healing, tissue adhesion, and potent antibacterial activity against both *E. coli* and *S. aureus* [120]. These hydrogels accelerate wound healing while monitoring patient movements.

All-natural conductive supramolecular hydrogels represent another innovative direction. Systems like the GT5-DACD2-B hydrogel, formed through Schiff base reactions between gelatin and dialdehyde-β-cyclodextrin, incorporate fusidic acid (FA) via host–guest inclusion for inhibition of *S. aureus* proliferation [121]. Enhanced with tannic acid for antibacterial synergy and borax for conductivity, these fully natural materials offer excellent biocompatibility while functioning as flexible sensors for motion, facial expression, and speech recognition.

#### 4.4.2. Bioresponsive Closed-Loop Systems

True innovation emerges when hydrogel–electronics integration enables autonomous, biomarker-responsive therapeutic actions. A groundbreaking example is the closed-loop patch (CLP) with biomimetic infection sensing, which detects bacterial hyaluronidase (HAase) secreted by pathogens like *S. aureus* [122]. The system uses an engineered hyaluronic acid hydrogel that degrades in response to HAase, changing the capacitance of interdigitated electrodes and triggering release of titanium hydride nanoparticles for sonodynamic antibacterial therapy. This “detect-and-treat” approach provides early infection warning before visible symptoms appear and significantly accelerates wound healing.

Multi-stimuli responsive hydrogels offer sophisticated control over therapeutic delivery. A four-in-one pH/glucose-responsive engineered hydrogel (PPy&L-arg@PVA-TSPBA) utilizes dynamic boronate ester bonds that cleave in response to the high glucose and low pH characteristic of infected diabetic wounds [123]. This triggers controlled release of antioxidant polypyrrole (PPy) and pro-angiogenic L-arginine, while the photothermal properties of PPy enable near-infrared activated bacterial elimination. The system synergistically addresses bacterial infection, oxidative stress, chronic inflammation, and impaired angiogenesis through a single integrated platform.

#### 4.4.3. Integrated Intelligent Platforms

Advanced integration extends to 3D architectures that combine structural support with sensing and therapeutic functions. A notable example is the 3D hybrid scaffold with nanofiber yarns embedded in injectable hydrogel, created by weaving aligned polyacrylonitrile/reduced graphene oxide nanofibers and encapsulating them in a Schiff base-crosslinked hydrogel [124]. This construct provides directional guidance for cell alignment, antibacterial properties, and conductive pathways for strain sensing—enabling simultaneous wound monitoring and promotion of healing. In addition, machine learning-assisted visual monitoring hydrogels like the GelMA and chitosan methacrylate (CMCSMA) hydrogel incorporate phenol red as a pH indicator, enabling smartphone-based colorimetric analysis [125]. Machine learning algorithms process the spectral signals to reliably assess wound pH, optimizing treatment management based on wound status.

The convergence of hydrogel and flexible electronics technologies has led to diverse functional systems for diabetic wound management, each with distinct mechanisms and applications. To systematically compare these emerging technologies, Table 1 categorizes representative flexible electronic devices based on their primary functions, technical approaches, and operating mechanisms. This functional comparison provides a structured overview of current research trends and technological innovations in the field.

## 5. Challenges and Future Prospects

The sophisticated functionalities envisioned for integrated hydrogel–flexible electronics systems present a stark contrast to the established standards of care in current clinical practice. The commercial market for diabetic wound management is dominated by passive, single-function hydrogel dressings. Products such as IntraSite™ Gel (Smith & Nephew, Watford, UK; modified carboxymethylcellulose for moisture retention and autolytic debridement), Purilon^®^ Gel (Coloplast, Humlebaek, Denmark; calcium alginate and carboxymethylcellulose sodium for exudate absorption), and antimicrobial variants like Silvercel^®^ (Cardinal Health, Dublin, OH, USA; alginate and carboxymethylcellulose hydrogel with ionic silver) are foundational in managing wound hydration and infection. However, these dressings operate on purely physicochemical principles without electronic components, lacking the capacity for real-time monitoring or feedback-controlled therapy. Few products that deeply and functionally integrates a therapeutic hydrogel matrix with flexible electronics for diagnostic–therapeutic synergy have yet reached the global medical market. Prototypes demonstrating such integration remain in preclinical or early clinical development. Bridging this gap and translating such integrated systems into clinical tools requires overcoming multifaceted challenges, which are analyzed in the following sections.

One of the foremost limitations lies in material stability and functional durability, which critically influence overall system performance. Many functional hydrogels are prone to degradation or loss of bioactivity under ambient conditions, with particular concern surrounding the stability of growth factors and natural antioxidants. These vulnerabilities require urgent resolution. Similarly, flexible electronic components are susceptible to signal drift, interface delamination, and mechanical fatigue during extended use, which can compromise continuous functionality and data fidelity. Future research should prioritize the development of advanced materials endowed with self-healing properties, dynamic responsiveness, and multifunctional capabilities. In parallel, optimization of interface architectures and micro-adhesion strategies will be essential to improve system robustness and operational longevity.

In addition to material-related constraints, the complexity associated with multifunctional integration imposes substantial demands on fabrication processes and system reliability. As the number of functional modules increases, system architectures become more intricate, leading to fabrication workflows that are both cumbersome and cost-intensive. These factors contribute to reduced stability and scalability. A key engineering challenge in this context is achieving a balance between multifunctionality and structural simplification, while promoting process standardization. Promising strategies to address this challenge include the adoption of modular design principles, the application of microfabrication technologies, and the utilization of programmable materials. These approaches may pave the way for future platforms characterized by high integration, enhanced reliability, and streamlined manufacturing.

Beyond material and engineering considerations, individual variability and the demand for treatment precision present substantial challenges to the integration of hydrogel–flexible electronics systems. Patients with diabetes exhibit considerable heterogeneity in wound types, healing dynamics, and physiological responses, making standardized treatment protocols insufficient to meet personalized clinical needs. To address this complexity, integrated systems must possess adaptive capabilities that allow dynamic adjustment of therapeutic strategies in response to real-time wound conditions, thereby enabling truly individualized care. Future directions may involve the incorporation of three-dimensional printing technologies in combination with biofeedback mechanisms to construct customizable treatment platforms, enhancing both clinical adaptability and therapeutic efficacy.

Another critical barrier to clinical translation lies in the absence of standardized validation protocols and regulatory frameworks. Most current research remains confined to in vitro models or animal studies, lacking systematic clinical validation and long-term follow-up data. Simultaneously, the regulatory classification of integrated systems remains ambiguous, as they do not conform to conventional definitions of pharmaceuticals or standalone medical devices. This ambiguity underscores the need for the development of novel evaluation criteria and approval pathways. Establishing robust regulatory frameworks and standardized clinical trial protocols will be essential for advancing industrial translation and ensuring patient safety.

Artificial intelligence-assisted diagnostic and therapeutic systems are anticipated to play a pivotal role in the evolution of next-generation convergent platforms. By integrating data acquired through flexible electronics with AI algorithms, it will be possible to achieve automated wound status recognition, personalized treatment planning, and predictive prognosis modeling. These capabilities have the potential to transform wound care into a domain of intelligent, data-driven healthcare. The development of personalized treatment platforms will become a central research focus, aiming to design customized systems tailored to individual wound types, metabolic profiles, and immune characteristics, thereby achieving precision therapy and efficient tissue repair.

Continued progress in novel materials and interface engineering is anticipated to further enhance system performance. The development of hydrogel materials with multiresponsive behavior, self-healing capacity, and intrinsic biological activity, along with the optimization of interface adhesion and signal transmission between hydrogels and electronic components, will be critical for ensuring long-term operational stability. The advancement of this field will be driven by interdisciplinary collaboration across materials science, electronic engineering, clinical medicine, and data science. Such integration has the potential to establish comprehensive research platforms and clinical translation pathways, accelerating the transformation of experimental innovations into practical medical applications.

In conclusion, while substantial progress has been achieved in the integration of functional hydrogels with flexible electronics, widespread clinical adoption will require coordinated advancement across multiple dimensions. These include material innovation, system-level integration, intelligent algorithm development, and regulatory and clinical translation. Future research should prioritize the enhancement of system stability, the incorporation of intelligent decision-making frameworks, and the establishment of rigorous clinical validation protocols. The ultimate goal is to facilitate the seamless transition of these technologies from laboratory research to bedside application.

## 6. Conclusions

The integration of functionalized hydrogels with flexible electronics represents a convergent paradigm at the intersection of materials science and biomedical engineering, offering a novel technological pathway for the intelligent management of diabetic wounds. This paper presents a systematic review of the fundamental principles underlying hydrogel design, the material systems employed, and the strategies used to integrate flexible electronic devices. It further examines the engineering challenges associated with interfacial integration and material construction, and evaluates the practical performance and clinical potential of these hybrid systems through representative application examples.

Through the combination of functional molecular modification and the deliberate design of dynamic response mechanisms, hydrogel materials can be endowed with a wide range of therapeutic functions, including antibacterial, antioxidant, and pro-angiogenic properties. Flexible electronic devices, equipped with high-sensitivity sensing and remote interaction capabilities, enable real-time monitoring and intelligent feedback of wound microenvironments. The synergistic integration of these two components has been shown to improve treatment precision and responsiveness, thereby driving a shift in wound care from traditional passive dressings toward active diagnostic and therapeutic platforms.

Despite notable progress, several challenges remain, including issues related to material stability, the complexity of system integration, personalized adaptability, and the lack of established clinical translation pathways. Addressing these challenges will require sustained multidisciplinary collaboration to support the development of advanced materials, the incorporation of intelligent algorithms, and the creation of robust clinical validation frameworks. Such efforts are essential to facilitate the transition from laboratory prototypes to clinically deployable products.

The fusion of functional hydrogels with flexible electronics constitutes a rapidly emerging field, propelled by technological innovation and growing clinical demand. Its potential to serve as a foundational platform for diabetic wound management is considerable, with implications for advancing personalized, intelligent, and precision medicine. Continued research in this area is expected to yield dual benefits: improving patient quality of life and establishing scalable technological paradigms for the treatment of chronic diseases. The emergence of these integrated systems signals the beginning of a new era defined by the deep convergence of smart biomaterials and flexible electronic technologies.

## Figures and Tables

**Table 1:** Functional Comparison of Flexible Electronic Devices for Diabetic Wound Management.

Functions	Technical Subcategory	Mechanisms	References
Monitoring and Sensing	Single-parameter sensing	Temperature mapping	[106]
		pH monitoring	[105]
	Multi-parameter sensing	Microfluidic sensing of temperature, pH and uric acid	[103]
		Microgel sensing of temperature, pH and glucose	[104]
Active Therapy	Electrical stimulation	Self-powered electrical stimulation through biomechanical energy conversion	[87,126]
		Programmable electrical stimulation with parameter control	[117,127]
		TENG-based electrical stimulation from mechanical motion	[115]
	Controlled Drug delivery	Electrically triggered on-demand drug release using conductive hydrogels	[116]
		Minimally invasive transdermal delivery via bioinspired microneedles	[118]
Integrated Theranostics	Conductive/Responsive Hydrogel Architectures	High-conductivity hydrogel platform combining sensing and stimulation	[119,120,121,128]
	Bioresponsive Closed-Loop Systems	Closed-loop infection response with automated detection and treatment	[122]
		Glucose–pH dual-responsive release of therapeutic agents	[123]
	Integrated intelligent platforms	3D hybrid scaffold providing structural guidance and real-time monitoring	[124,129]
		Machine learning-assisted visual wound monitoring and assessment	[125]

## Data Availability

No new data were created or analyzed in this study. Data sharing is not applicable to this article.

## Abbreviations

The following abbreviations are used in this manuscript:

AGEs	Advanced glycation end products
CPBA	4-Carboxyphenylboronic acid
EGCG	(-)-Epigallocatechin gallate
GelMA	Methacryloyl gelatin
PBA	Phenylboronic acid
PVA	Polyvinyl alcohol
PVDF	Polyvinylidene fluoride
ROS	Reactive oxygen species
VEGF	Vascular endothelial growth factor
